# Hybridisation and diversification in the adaptive radiation of clownfishes

**DOI:** 10.1186/s12862-014-0245-5

**Published:** 2014-11-30

**Authors:** Glenn Litsios, Nicolas Salamin

**Affiliations:** Department of Ecology and Evolution, Biophore, University of Lausanne, 1015 Lausanne, Switzerland; Swiss Institute of Bioinformatics, Génopode, Quartier Sorge, 1015 Lausanne, Switzerland

**Keywords:** Syngameon, Speciation, Diversification, Cytonuclear discordance, Anemonefish

## Abstract

**Background:**

The importance of hybridisation during species diversification has long been debated among evolutionary biologists. It is increasingly recognised that hybridisation events occurred during the evolutionary history of numerous species, especially during the early stages of adaptive radiation. We study the effect of hybridisation on diversification in the clownfishes, a clade of coral reef fish that diversified through an adaptive radiation process. While two species of clownfish are likely to have been described from hybrid specimens, the occurrence and effect of hybridisation on the clade diversification is yet unknown.

**Results:**

We generate sequences of three mitochondrial genes to complete an existing dataset of nuclear sequences and document cytonuclear discordance at a node, which shows a drastic increase of diversification rate. Then, using a tree-based jack-knife method, we identify clownfish species likely stemming from hybridisation events. Finally, we use molecular cloning and identify the putative parental species of four clownfish specimens that display the morphological characteristics of hybrids.

**Conclusions:**

Our results show that consistently with the syngameon hypothesis, hybridisation events are linked with a burst of diversification in the clownfishes. Moreover, several recently diverged clownfish lineages likely originated through hybridisation, which indicates that diversification, catalysed by hybridisation events, may still be happening.

**Electronic supplementary material:**

The online version of this article (doi:10.1186/s12862-014-0245-5) contains supplementary material, which is available to authorized users.

## Background

Hybridisation has long been considered as a process reducing genetic diversity through introgression [1]. Thus, the effect of hybridisation on species diversification should, if anything, be negative due to the potential reduction in fitness of the hybrids. However views are changing as many evolutionary radiations have now documented cases in which hybridisation plays a key role in promoting species diversification. Common examples of hybridisation inducing diversification can be found in the Galapagos finches [2], Hawaiian *Lapaula* crickets [3] and Rift Lakes cichlids [4,5]. Building on those evidences and others, Seehausen proposed that hybridisation may promote speciation and adaptive radiation by generating new genetic and phenotypic variation that can be the target of selection [6]. With more and more cases of hybrid speciation being documented [7], the main question is now shifting towards understanding how evolutionary radiations are facilitated or even catalysed, rather than prevented, by hybridisation [8].

During adaptive radiation, available ecological niches are filled by the diversifying species [9]. In the classical view, this process occurs through specific phenotypic adaptations that allow the constituent species to take advantage of the diversity of ecological niches [10]. Hybridisation between incipient species has been proposed as a mechanism that is able to rapidly create new phenotypic combinations suitable for further occupancy of untapped niches [6]. Indeed, transgressive segregation can produce individuals with novel phenotypes reaching beyond the possibilities of the parental populations [11,12]. For example, the complementary action of genes regulating mineral ion uptake allowed hybrids of species of *Helianthus* sunflowers to survive in salt marshes while none of the parent species are salt tolerant [13]. Such an event can create new species very rapidly, especially when the newly formed hybrids are ecologically distinct from the two parent species [14]. For instance, as little as 10 to 60 generations are needed for recombination to no longer reduce the size of parental linkage blocks in *Helianthus*, which is a clear sign of genome stabilisation in the hybrids [15]. This mechanism is nevertheless dependent on the availability of untapped ecological niches. Indeed, the frequency of hybridisation events leading to speciation will decrease after the initial burst of adaptive radiation because of the saturation of the niche space [6]. While the theoretical grounds defining the potential role of hybridisation as an important component of species diversification have been laid, empirical evidence is needed to better illustrate the effects of this factor during adaptive radiations.

Several methods exist to identify putative hybridisation events in evolutionary radiations (reviewed in [6]). Discordance between phylogenetic trees inferred with either cytoplasmic (chloroplast or mitochondrial) or nuclear DNA can suggest the occurrence of hybridisation. This is possible because phylogenetic trees based on cytoplasmic information will show the evolutionary history of females while nuclear DNA will illustrate that of the parental allele that has been fixed. Moreover, a tree-based method (the homoplasy excess test; HET) has been proposed to identify putative hybrid taxa and likely parental lineages [6]. While DNA sequences of cytoplasmic or nuclear origin are now readily available for a growing number of species, these approaches have only been used scarcely (e.g. [16]). Given the potential importance of hybridisation for species diversification, there is a need to study new cases such as comprehending more fully the interactions between hybridisation and speciation.

In this context, we study hybridisation patterns in the clownfishes (or anemonefishes; Pomacentridae), a monophyletic clade which maintains mutualistic interactions with sea anemones [17]. This behaviour is the key innovation that promoted the adaptive radiation of clownfishes as species segregated among specific combinations of potential host species and habitat, each time matching their phenotype to the environment [18]. While this process of ecological speciation [9] could alone be responsible for the extant diversity of clownfish species, evidence suggests that hybridisation may have occurred during the evolution of the group. Indeed, a recent phylogenetic tree of the clade based on nuclear markers showed several discordant nodes with previous phylogenetic trees reconstructed mostly from mitochondrial markers [19]. Moreover, interspecific pairs have been observed in the wild and two species, *Amphiprion leucokranos* and *A. thiellei*, were likely described from hybrid material [17,20]. This indicates a potential history of hybridisation in the group. However, whether it impacted on the diversification of clownfishes is yet unknown.

To resolve this issue, we analyse a dataset of mitochondrial and nuclear DNA sequences of clownfish species to assess cyto-nuclear discordances and highlight possible ancient hybridisation events. Furthermore, we use the HET approach to identify species of hybrid origin. Finally, we investigate potential hybrids among clownfish individuals showing hybrid phenotypic characteristics and use molecular cloning to assess their status and putatively identify the parental species.

## Results

We define from phylogenetic inference eight monophyletic groups of species to facilitate the interpretation of our results (Table 1). The clade names are based on classical clownfish taxonomy that describes 6 species complexes [21].

**Table 1:** **Clades of clownfishes used in this study**

**Clade name**	**Species**
*percula*	*A. ocellaris, A. percula, P. biaculeatus*
Australian	*A. akindynos, A. mccullochi*
*akallopisos*	*A. akallopisos, A. perideraion, A. pacificus, A. sandaracinos*
*ephippium*	*A. frenatus, A. ephippium, A. rubrocinctus, A. melanopus, A. barberi*
*polymnus*	*A. sebae, A. polymnus*
*clarkii*	*A. clarkii, A. tricinctus*
Indian	*A. bicinctus, A. omanensis, A. chagosensis, A. latifasciatus, * A. nigripes, A. allardi, A. chrysogaster, A. fuscocaudatus*

The table gives the list of species included in each clade. *A*. stands for *Amphiprion*, *P*. for *Premnas*. Not included are *A. chrysopterus* and *A. latezonatus*, which are monospecific lineages and *A. leucokranos* and *A. thiellei*, which have likely been described from hybrid specimens. *Exemplars of *A. fuscocaudatus* have never been sequenced. We hypothetically placed this species in the Indian clade because it is the most parsimonious solution regarding the biogeography of clownfish species [19].

### Discordance between phylogenetic hypotheses

**Figure 1:**
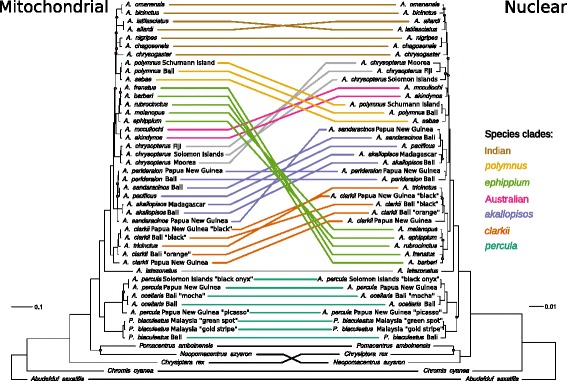
***Cytonuclear incongruence.*** Majority-rule consensus tree with all compatible groups for mitochondrial (on the left) and nuclear (on the right) datasets. Red dots indicates nodes having Bayesian posterior probabilities lower than 0.95. Links are drawn to highlight the topological differences. Colours correspond to species clades (see Table 1) as follows: black: outgroups, turquoise: *percula*, grey: *Amphiprion latezonatus* and *A. chrysopterus* (monospecific lineages), orange: *clarkii*, blue: *akallopisos*, rose: Australian, green: *ephippium*, yellow: *polymnus*, brown: Indian. A coloured legend, which repeats this information for the major clades, is located at the right of the figure.

**Figure 2:**
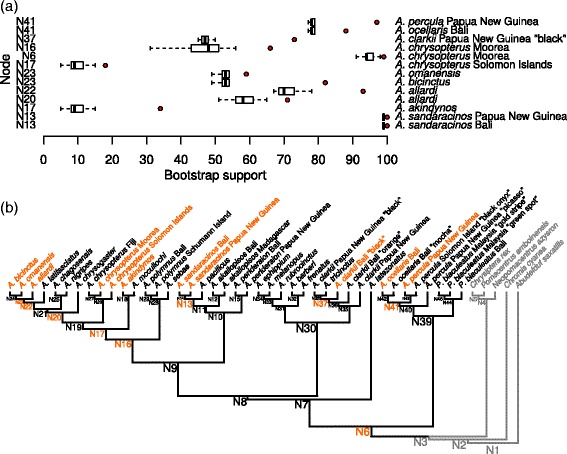
***Homoplasy excess test.*** Panel **(a)** shows the distribution of the bootstrap support for each node which showed a homoplasy excess during the removal analysis. Each boxplot shows the null distribution of bootstrap values for the node and the red dot shows the bootstrap value of the node when the taxon of interest (name on the right) has been removed. Nodes names as given on the left correspond to that of panel **(b)** which shows the consensus tree of the nuclear phylogeny. There, nodes and taxa which showed up as outliers in the analysis have been coloured in orange. Species used as outgroup are shown in grey. The size of node labels has been altered to facilitate reading.

The two consensus phylogenetic trees that we obtain show an overall good support with only several recent splits showing low posterior probabilities (Figure 1). Major clades appear in both mitochondrial and nuclear phylogenetic trees. Despite the fact that the two datasets show well resolved topologies, the relative position of the main clades is different between the two analyses (Figure 1, see Additional files 1 and 2 for a complete illustration of node support). The organisation of the *percula* clade at the base of the clownfish tree and the position of *A. latezonatus* is congruent between the two datasets. However, the Australian group and *A. chrysopterus* intercalate between the *polymnus* and Indian groups in the nuclear dataset. We identify two mitochondrial lineages in *A. sandaracinos* with a posterior probability of 1, but both samples are clustered in the nuclear phylogenetic tree. The *ephippium* clade, which is the sister group of the *clarkii* clade in the nuclear phylogenetic tree, becomes sister to the Australian clade with the mitochondrial dataset. This suggest the existence of a hybridisation event at the time of node 8 (Figure 2). Other studies showed that a main upward shift in speciation rate occurred at this specific node [22,23].

### Hybrid signal

We recover an excess of homoplasy at nine nodes of the clownfish phylogenetic tree (Figure 2). Removing either *A. ocellaris* from Bali or *A. percula* from Papua New Guinea increases the BS of Node 41 (Figure 2), which suggests genetic exchange between members of the *percula* clade. We find that the “black” specimen of *A. clarkii* collected in Bali likely originated from hybridisation between other members of the *clarkii* group (node 37, Figure 2). We measure further increase of BS at three different nodes (6, 16 and 17, Figure 2) when individuals of *A. chrysopterus* from either Moorea or the Solomon Islands are removed. Moreover, the BS of node 17 increases when *A. akindynos* is removed, which suggests several hybridisation events between *A. chrysopterus* and species of the Australian and *polymnus* groups occurring around the timeframe of node 16 and 17. We also find evidence of genetic exchanges between members of the Indian group at nodes 20, 22 and 23 (Figure 2). Finally, we identify gene exchange between the two *A. sandaracinos* individuals at node 13 and *A. pacificus*, but the effect on bootstrap values is relatively small.

### Identification of recent hybrid parental species

**Figure 3:**
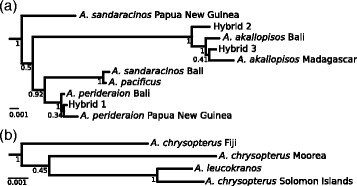
***Mitochondrial phylogeny of hybrid individuals*** **.** Majority-rule consensus tree with all compatible groups for mitochondrial sequences with the inclusion of hybrid specimens in the analysis. Panel **(a)** shows a close-up on the topological position of hybrid specimens 1 to 3 and panel **(b)** of *A. leucokranos*. The topology of the rest of the phylogeny (not shown) is as in Figure 1.

**Figure 4:**
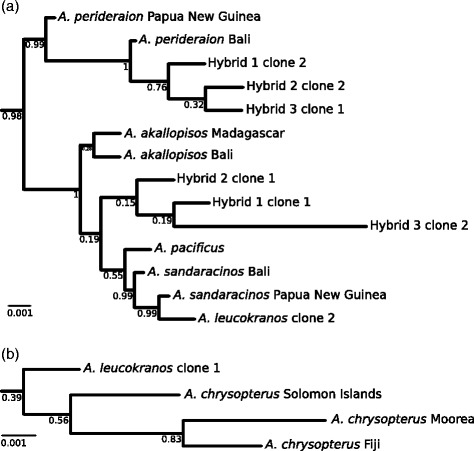
***Nuclear phylogeny of hybrid cloned sequences.*** Majority-rule consensus tree with all compatible groups for nuclear sequences with the inclusion of hybrid specimens in the analysis. The two panels **(a & b)** are close-ups on the topological position of the cloned sequences. The topology of the rest of the phylogeny (not shown) is as in Figure 1.

**Figure 5:**
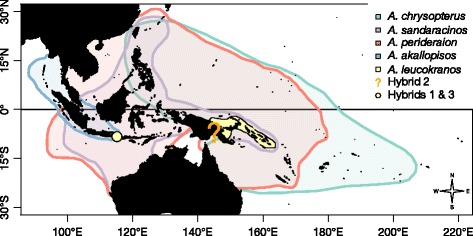
***Distribution of potentially hybridising species*** **.** Species distribution following [17], of species likely implicated in hybridisation events. The localities of the sequenced hybrid individuals are shown by yellow dots or area when known. The precise geographical origin of the hybrid 2 individual is unknown, the question mark sign indicates the general area where it was collected.

We infer the phylogenetic trees of mitochondrial (Figure 3) and nuclear (Figure 4) markers for all sampled clownfish species augmented with the additional sequences of the supposed hybrid individuals. The cloning procedure allowed us to separate the two parental BMP4 sequences of each hybrid individual analysed (clones 1 and 2 in Figure 4). As expected, we find that the BMP4 sequences from those hybrid individuals that were cloned do not cluster by individual species but are separated among parental clades (Figure 4). We deduced the maternal origin of the putative hybrids with the mitochondrial phylogenetic tree. We find that both hybrids 2 and 3 inherited their mitochondrial genes from *A. akallopisos*. The plastid sequence of hybrid 1 is similar to *A. perideraion* and *A. leucokranos*, as already proposed [20], derives from *A. chrysopterus*. Moreover, in the mitochondrial tree, *A. leucokranos* clusters with *A. chrysopterus* sampled from the Solomon Islands, the origin of *A. leucokranos* (Figure 5). The maternal origin of the four hybrid individuals is highly supported in the phylogenetic tree with posterior probabilities of 1 on the respective ancestral nodes (Figure 3). The nuclear tree confirms those results and each individual has one of the cloned sequence that clusters with the same species as in the mitochondrial tree (Figure 4). The position of the other clone provides information on the paternal origin of the hybrids. *A. leucokranos* clusters with high posterior probability with *A. sandaracinos* and hybrid 1 and 3 both cluster with *A. perideraion*. The other hybrid individual (hybrid 3) clusters with any of *A. pacificus*, *A. sandaracinos* and *A. akallopisos*, but low posterior probabilities forbid any definitive conclusion. Nevertheless, we do not find clear evidence of recombination events amongst the cloned sequences, suggesting that we sequenced individuals deriving from recent hybridisation events.

## Discussion

Our results show instances of cyto-nuclear incongruence in the clownfish phylogenetic tree, which suggests several hybridisation events. The most ancient of those events occurred in the same time frame as a node that was identified twice independently as the highest increase in diversification rate happening in the damselfish family [22,23]. This evidence suggests a relationship between this ancient hybridisation event and the main burst of diversification of the adaptive radiation of clownfishes. Moreover, we identify nodes with an excess of homoplasy that can be attributed to more recent hybridisation events. Finally, we identify the parental lineages of four hybrid individuals, showing that hybridisation still occurs within clownfishes. This evidence suggests that the diversification of the clownfishes has been likely fuelled by hybridisation events that created novel phenotypes and eventually new species in a manner similar to the well-known East African cichlids.

### Past hybridisation

We reconstruct separately the phylogenetic trees of the mitochondrial (three markers) and nuclear (seven markers) genomes. Most nodes show high posterior probabilities, yet, several shallow nodes of our mitochondrial and nuclear trees are only weakly supported. This can originate from the lack of phylogenetic informative sites between closely related species. Previous studies of phylogenetic relationships of the *Pomacentridae* inferred by mitochondrial or nuclear markers, all support the monophyly of the clownfish as well as the existence of the main clownfish clades [18,23-25]. The topology obtained with the mitochondrial data corresponds to these published phylogenetic trees.

We find several deep cytonuclear discordances in the clownfish. The relative position of the *ephippium* clade changes radically between the two datasets, which suggests the occurrence of hybridisation in the timeframe of node 8 (Figure 2). While this node is not basal to all clownfishes, it is ancestral to 24 of the 28 species (30 described species minus *A. leucokranos* and *A. thiellei* which were likely described from hybrid material [20]). This clade represents a major shift in diversification rate with a 2.5-fold increase compared to the diversification rate of Pomacentridae [22,23]. The pattern of concurrent increase in rate of diversification and occurrence of hybridisation fits into the framework of the syngameon hypothesis [6], where hybridisation events preludes an acceleration of ecological differentiation and diversification rate. Moreover, this result is robust regarding node support, as the relationship between the *clarkii* and the *ephippium* clade is itself highly supported (see Additional file 1).

We find paraphyletic mitochondrial genotypes in *A. sandaracinos*. The fact that the two *A. sandaracinos* individuals cluster with high support in the nuclear dataset suggests a recent hybridisation event implying a mitochondrial genotype that belongs to an unknown lineage of the *akallopisos* clade. Results of the HET suggest the ongoing occurrence of genetic exchanges between *A. sandaracinos* and *A. pacificus* (node 13, Figure 2). The two species are phenotypically very similar and it is possible that a third cryptic species having the mitochondrial profile of the *A. sandaracinos sample* from Papua New Guinea exists. The remaining topological inconsistencies of the *polymnus* clade are not well supported as shown by the Bayesian posterior probabilities of their respective nodes on the mitochondrial phylogeny. This discrepancy may be artifactual and is thus not further discussed.

### Hybridisation and adaptive radiation

Our results highlight the existence of a main hybridisation event at the base of the *Amphiprion* crown group (node 8, Figure 2). This node has been previously linked with a burst of diversification rate in the clownfish clade [22,23] and may also be mainly responsible for the increase in rate of morphological evolution found in the group [18]. In contrary, we did not detect traces of ancient hybridisation in the *percula* group which is species poor relative to the *Amphiprion* crown group. One recent hybridisation event likely occurred around node 41 (Figure 2) between *A. percula* and *A. ocellaris*. The distribution of those two species is mostly allopatric, but they share a common boundary and may overlap at some locations [26] possibly leading to gene exchange. Yet, despite similar behaviour and ecology, the species of the *percula* clade seemingly failed to radiate [19]. The fact that the clade deriving from a major hybridisation event is the only one to show patterns of rapid speciation supports the view that hybridisation can promote diversification during adaptive radiation. Actually, hybridisation appears to be one of the key factors explaining the adaptive radiation of the clownfishes.

### Recent hybridisation events

We identify the parental species of four putative hybrids, one being *A. leucokranos*, which is currently described as a species. Since their taxonomic descriptions, the species status of *A. leucokranos* and *A. thiellei* has been questioned [20]. Indeed, heterospecific pairs have been observed in the wild and fish with similar phenotype can be produced in aquaria by hybrid crosses [17]. Nevertheless, evidence of *A. leucokranos* pairs also exist [27], tentatively proposing that we may be witnessing hybrid speciation in progress. Yet, demonstrating this ongoing process would require more data than available here. It is thus more parsimonious to conclude at the moment, that *A. leucokranos* is an invalid species.

We separate BMP4 sequences of each parental chromosome through molecular cloning and identify the putative parental species of *A. leucokranos* as *A. chrysopterus* and *A. sandaracinos*. This is consistent with field observations [20]. The hybrid individuals that we sequenced are all likely recent hybrids (possibly F1) because the two clones can always be clearly separated and cluster with different putative parental taxa. This suggests that genomic rearrangement occurring during reproductive events did not yet occur.

The special social structure of clownfish may put some behavioural constraints on hybridisation. Indeed, all clownfish species are protandrous hermaphrodites and live in social groups with size-based dominance hierarchy. The female is the largest and dominant individual, the male the second largest, and usually smaller non-breeding individuals follow, decreasing in size relative to their hierarchical position [28]. In certain cases [29-31], size differentiation allows smaller species to cohabit with a larger species in the same anemone. While reproduction of the smallest species is largely suppressed by the presence of the largest clownfish [32], species hybridisation cannot be fully excluded. In such instance, the size-based hierarchy ensure that the female of a hybrid pair will most probably always be the largest individual. This is what we observe in *A. leucokranos*. The parental origin of the three other hybrids does not allow for a definitive conclusion regarding this behavioural constraint. Indeed, the size difference between species of the *akallopisos* group is small and cannot be used as a diagnostic feature separating *A. akalloposis*, *A. pacificus and A. perideraion* [33]. It is thus likely that the behavioural constraint on hybridisation between those species is going to be weaker than between species with large size difference.

### Geography of recent hybridisation

Hybrid zones appear when two species share such margins and have the possibility to exchange genes. At the range margins, the probability of encountering a heterospecific mate is higher than that of meeting an individual of its own species whenever an individual moves into the range of the other species [34]. This will increase the probability of hybridisation events. As expected, *A. leucokranos* is distributed at the edge of the two parental distributions and hybrids 1 and 3 were found at the eastern edge of the range of *A. akallopisos* (Figure 5). Further genetic sampling would be necessary to better define those potential hybrid zones. This would be of particular interest given the evolutionary significance of hybridisation in animals, as exemplified by the importance of exogenous selection on contemporary hybrid zones [35].

## Conclusion

Hybridisation can impact speciation in many ways. Our analyses show that in the case of the adaptive radiation of the clownfishes, hybridisation events are linked with a burst in diversification rate. We observe that hybridisation has occurred throughout the evolutionary history of the group and is still happening. Further studies are required to measure the extant of ongoing hybrid speciation, for example in *A. leucokranos*. There, field observations coupled with experiments will allow to identify the conditions in which hybrids have a better fitness than their parents, illustrating the thight relationship linking species ecology and evolutionary processes.

## Methods

### Phylogenetic reconstruction

We used previously extracted DNA from 41 individuals (see [19] for details; Additional file 3: Table S1) representing 27 species of clownfish to analyse the evolutionary history of mitochondrial genes. We amplified parts of the ATP86, CytB and 16S markers via polymerase chain reaction (PCR) with a 25 μl reaction mixture containing ~50 ng of DNA template, 2.5 μL GoTaq buffer, 3 μL dNTPs (2.5 μM), 1 MgCl2 (25 μM), 1 μL of both forward and reverse primer and 0.3 μL of Taq polymerase (GoTaq DNA Polymerase, Promega, Madison, WI). Primer sequences and PCR cycles were set as in [24] for ATP86 and CytB, and [25] for the 16S. We purified the amplification products with the QIAquick PCR Purification Kit (Qiagen, GmbH, Germany). We sequenced the purified products forward and reverse strands with the Big Dye 3.1 Terminator cycle sequencing kit (Applied Biosystems, Foster City, CA), according to the manufacturer’s instructions, and separated the products on an ABI Prism 3100 genetic analyser (Applied Biosystems, Foster City, CA). We assembled a nuclear dataset from sequences generated in a previous study [19]; Additional file 3: Table S1). The nuclear dataset is 6,679 base pairs long and comports seven genes (BMP4, Glyt, Hox6, RAG1, S7, Svep, Zic1). We used MAFFT 6.864b [36] with default settings to align each mitochondrial and nuclear gene individually. We verified each alignment visually and concatenated the mitochondrial and nuclear datasets in two separate super-matrices with SequenceMatrix [37]. We estimated the mitochondrial and nuclear phylogenetic relationships of clownfish species with MrBayes 3.2.1 [38]. The parameters of the nucleotide substitution process for each gene in the mitochondrial and nuclear data sets were sampled across the GTR model space during the Bayesian MCMC [39]. This approach has the advantage of removing the need of a priori testing for a specific model of substitution (e.g. [40]). We performed, for each dataset, two independent runs of MrBayes with four parallel MCMC chains, each 10 million generations long, and sampled model parameters every 1000 generations. We checked for optimal parameter convergence using the ‘sump’ command and by visualising the trace files and the ESS values of the MCMC chains using Tracer v 1.5 [41]. After the removal of 25% of the trees as burn-in, we merged the two independent runs to generate a majority-rule consensus tree for both mitochondrial and nuclear dataset that included all compatible groups.

### Hybrid signal

We assessed cytonuclear discordance by comparing visually the topology of both consensus phylogenetic trees. This step was facilitated by the use of the ”cophyloplot” function available in the R package Ape [42]. We further applied the framework of the HET on the nuclear dataset only [6]. Hybrid taxa are intermediate to the parental species when considering their nuclear genome, but they share identical alleles at each individual locus with only one parent [43]. When included in a phylogenetic tree based on a matrix of concatenated nuclear markers, a hybrid taxon will introduce an excess of homoplasy due to the conflicting phylogenetic signal present in the corresponding gene tree topologies in the clades containing the two parental species. Thus, the removal of a putative hybrid should reduce conflicts in the phylogenetic tree and result in increased support values for the nodes that includes the parental species [6]. While AFLP have initially been proposed as input data for the HET, it is conceptually similar to use the alignments of several nuclear genes. The only drawback of using sequence data is a reduction in power due to the lower amount of data and less independence between sites. Yet, by increasing the number of genes studied, this should not be a major issue. We implemented this test in R 3.0.1 [44] and used RAxML 7.3.5 [45] to perform the phylogenetic inference and bootstrap support (BS) using the ‘GTRGAMMA’ model of nucleotide substitution and specified a different partition for each gene in the dataset. For a species *i*, we first removed it from the complete concatenated alignment of nuclear data. We used this reduced data set lacking species *i* to build a phylogenetic tree and estimate node support with 100 bootstrap replicates. Second, we generated a null distribution of BS for each node of the consensus phylogenetic tree by removing at random a species *j* that is different from the focal species *i*, and inferred, again, the phylogenetic tree and BS. This was replicated 100 times. Third, we compared the BS for each node obtained by the reduced dataset lacking the focal species *i* to those of the null distribution. We identified outlier values of BS when the BS of a node fell above the highest value still within 1.5 interquartile range of the upper quartile of the null distribution [16,46]. This represent a case where the focal species *i* is of hybrid origin which has the effect of reducing drastically the BS of the node ancestral to the parental species. Finally, this process was carried on for each taxon included in the phylogeny. At first, we performed this analysis using single species removals, but then extended the framework to monophyletic pairs, triplets and so on, of species (*n* up to 8) to account for hybrid species which may have already further diversified into several descendants. Each time, we removed *n* random species from the dataset and generated a new null distribution of BS where *n* random species were removed from the alignment.

### Molecular cloning of recent hybrids

We obtained, through an aquarium fish importer, samples from three unidentified clownfish specimens that displayed hybrid characteristics such as aberrant colouration or shape. Hybrid 1 and 3 were collected in Bali, Indonesia, while hybrid 2 originated from Papua New Guinea. We followed the protocol described above to sequence, for each new sample, all three mitochondrial markers and the nuclear gene BMP4. We further isolated from those three individuals and a sample of *A. leucokranos* (a species that may have been described form hybrid individuals [20]), putative parental sequences of BMP4 using molecular cloning. We cloned the purified PCR products of the BMP4 gene into the pTZ57R/T vector using the InsT/Aclone PCR Product Cloning Kit (Fermentas, Vilnius, Lithuania). Then, we amplified up to 12 clones for each individual with the M13 forward and reverse primers to ensure that we sequenced all existing variants. We purified and sequenced the PCR products as above. We ensured, by checking visually all chromatograms, that no sequence showed double peaks, which would indicate heterozygous sites and thus a failure of the cloning procedure. We assembled identical clones in Geneious 6 (Biomatters), expecting to recover two different sequences (one for each parent) if the individual is truly a hybrid, or only one if it is not the case. We aligned the mitochondrial and nuclear sequences of the hybrid individuals to the two super-matrices with MAFFT [36]. Finally, we expected clones to cluster with the sequences of their parental species. We identified the putative parents of the individuals by reconstructing the phylogenetic trees of both super-matrices using MrBayes 3.2.1 in the same manner as described above. All newly generated sequences have been deposited in the EMBL database (accession numbers in Additional file 3).

### Availability of supporting data

DNA sequences are deposited in GenBank under the accession numbers found in Additional file 3. Multiple sequences alignments as well as phylogenetic trees are available in TreeBase, http://purl.org/phylo/treebase/phylows/study/TB2:S16652.

## Additional files

Additional file 1:
**Mitochondrial phylogeny with node support.**
Additional file 2:
**Nuclear phylogeny with node support.**
Additional file 3:
**Genbank accession numbers.**
