# Supplementary material for: Hybridisation and diversification in the adaptive radiation of clownfishes
Source: BMC Evol Biol. 2014 Nov 30;14:245. doi: 10.1186/s12862-014-0245-5 (PMC4264551; doi:10.1186/s12862-014-0245-5)

BMC Evolutionary Biology

Hybridisation and diversification in the adaptive radiation of clownfishes

Glenn Litsios and Nicolas Salamin

Additional File 3 Nuclear consensus phylogeny with node support. Code right of the species names correspond to the 'isolate' column of the additional file 1.

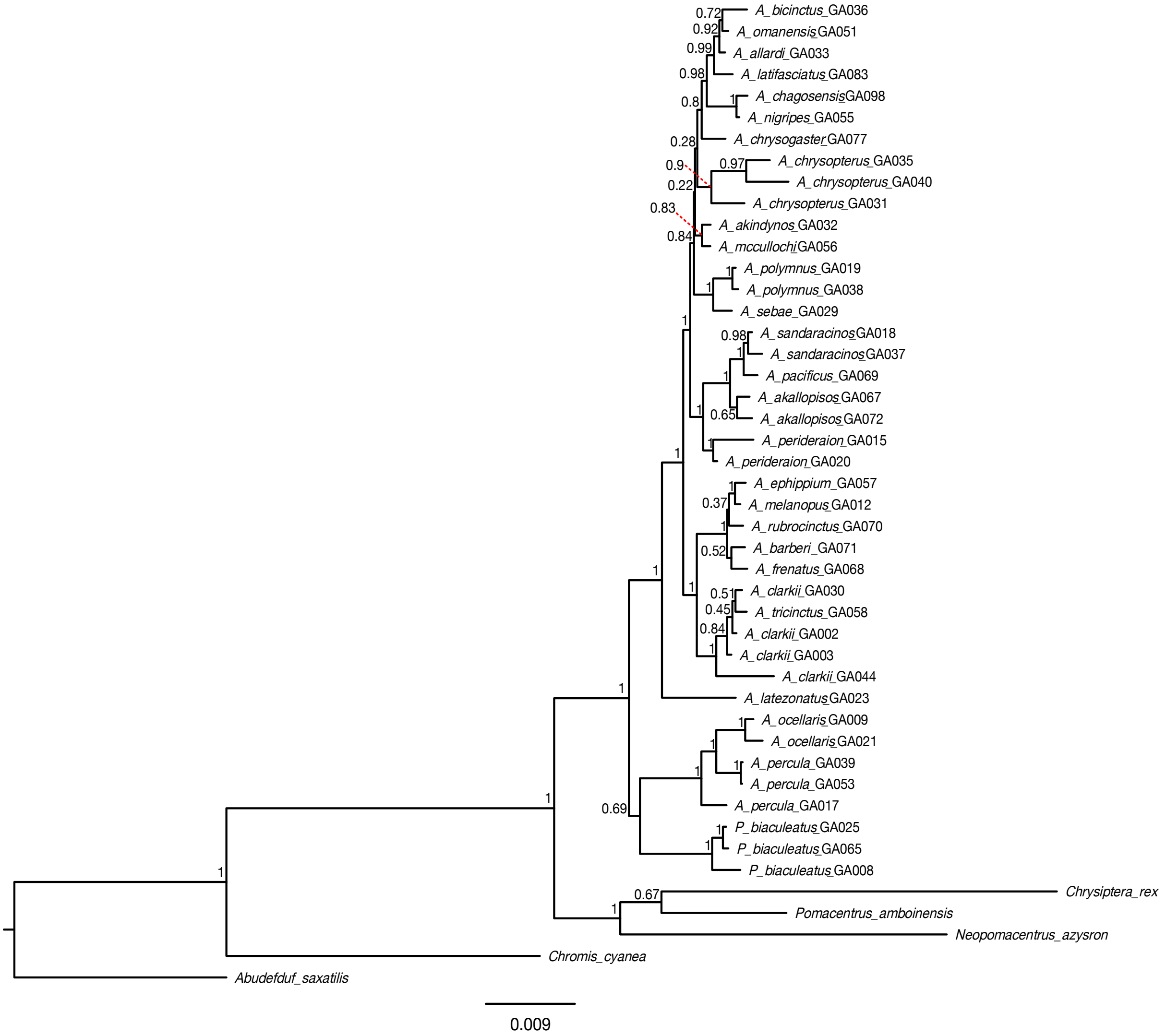

Supplement: Additional file 2: — Nuclear phylogeny with node support. [file 12862_2014_245_MOESM2_ESM.pdf]
