# Supplementary material for: Hybridisation and diversification in the adaptive radiation of clownfishes
Source: BMC Evol Biol. 2014 Nov 30;14:245. doi: 10.1186/s12862-014-0245-5 (PMC4264551; doi:10.1186/s12862-014-0245-5)
Supplement: Additional file 3: — Genbank accession numbers. [file 12862_2014_245_MOESM3_ESM.pdf]

*BMC Evolutionary Biology*

# Hybridisation and diversification in the adaptive radiation of clownfishes

Glenn Litsios and Nicolas Salamin

**Additional File 1** Genbank accession numbers and locality information (when known) for all sequences used in the study.

| Genus | Species | Isolate | Locality | clone | 16S | ATP86 | cytB | Bmp4 | Gylt | hox6 | s7 | svep | Rag1 | Zic1 |
|---|---|---|---|---|---|---|---|---|---|---|---|---|---|---|
| *Amphiprion* | *akallopisos* | GA067 | Bali | | KF264149 | KF264190 | KF264271 | KF264230 | KF264312 | KF774317 | KF774357 | KF774396 | KF264350 | KF264388 |
| *Amphiprion* | *akallopisos* | GA072 | Madagascar | | KF264150 | KF264191 | KF264272 | KF264231 | KF264313 | KF774318 | KF774358 | KF774398 | KF264351 | |
| *Amphiprion* | *akindynos* | GA032 | GBR | | KF264151 | KF264192 | KF264273 | KF264232 | KF264314 | KF774319 | KF774359 | KF774415 | KF264352 | KF264389 |
| *Amphiprion* | *allardi* | GA033 | Comoro Islands | | KF264152 | KF264193 | KF264274 | KF264233 | KF264315 | KF774320 | KF774360 | KF774401 | KF264353 | KF264390 |
| *Amphiprion* | *barberi* | GA071 | Fiji | | KF264153 | KF264194 | KF264275 | KF264234 | KF264316 | KF774321 | KF774361 | KF774418 | KF264354 | KF264391 |
| *Amphiprion* | *bicinctus* | GA036 | Red Sea | | KF264154 | KF264195 | KF264276 | KF264235 | KF264317 | KF774322 | KF774362 | KF774402 | KF264355 | KF264392 |
| *Amphiprion* | *chagosensis* | GA098 | Chagos | | KF819364 | KF819368 | KF819381 | KF819372 | | | KF774363 | KF774426 | KF819385 | |
| *Amphiprion* | *chrysogaster* | GA077 | Ile Maurice | | KF264155 | KF264196 | KF264277 | KF264236 | KF264318 | KF774323 | KF774364 | KF774424 | KF264356 | |
| *Amphiprion* | *chrysopterus* | GA031 | Solomon | | KF264156 | KF264197 | KF264278 | KF264237 | KF264319 | KF774324 | | KF774416 | KF264357 | KF264393 |
| *Amphiprion* | *chrysopterus* | GA035 | Fiji | | KF264157 | KF264198 | KF264279 | KF264238 | | KF774325 | KF774365 | KF774406 | | |
| *Amphiprion* | *chrysopterus* | GA040 | Moorea | | KF264158 | KF264199 | KF264280 | KF264239 | KF264320 | KF774326 | KF774366 | KF774407 | KF264358 | KF264394 |
| *Amphiprion* | *clarkii* | GA002 | Bali (black) | | KF264159 | KF264200 | KF264281 | KF264240 | KF264321 | KF774327 | KF774367 | KF774417 | KF264359 | KF264395 |
| *Amphiprion* | *clarkii* | GA003 | Bali (orange) | | KF264160 | KF264201 | KF264282 | KF264241 | KF264322 | KF774328 | KF774368 | KF774410 | KF264360 | KF264396 |
| *Amphiprion* | *clarkii* | GA030 | PNG (melanistic) | | KF264161 | KF264202 | KF264283 | KF264242 | KF264323 | KF774329 | KF774369 | KF774412 | KF264361 | KF264397 |
| *Amphiprion* | *clarkii* | GA044 | PNG | | KF264162 | KF264203 | KF264284 | KF264243 | KF264324 | KF774330 | KF774370 | KF774411 | KF264362 | KF264398 |
| *Amphiprion* | *ephippium* | GA057 | Indonesia | | KF264163 | KF264204 | KF264285 | KF264244 | KF264325 | KF774331 | KF774371 | KF774420 | KF264363 | KF264399 |
| *Amphiprion* | *frenatus* | GA068 | Philippines | | KF264164 | KF264205 | KF264286 | KF264245 | KF264326 | KF774332 | KF774372 | KF774425 | KF264364 | KF264400 |
| *Amphiprion* | 'hybrid 2' | GA095 | Papua New Guinea | 1 | KF819367 | KF819370 | KF819383 | KF819374 | | | | | | |
| *Amphiprion* | 'hybrid 2' | GA095 | Papua New Guinea | 2 | | | | KF819379 | | | | | | |
| *Amphiprion* | 'hybrid 1' | GA096 | Bali | 1 | KF819365 | KF819371 | KF819384 | KF819375 | | | | | | |
| *Amphiprion* | 'hybrid 1' | GA096 | Bali | 2 | | | | KF819380 | | | | | | |
| *Amphiprion* | 'hybrid 3' | GA091 | Bali | 1 | KF819366 | KF819369 | KF819382 | KF819378 | | | | | | |
| *Amphiprion* | 'hybrid 3' | GA091 | Bali | 2 | | | | KF819376 | | | | | | |
| *Amphiprion* | *latezonatus* | GA023 | Australia | | KF264165 | KF264206 | KF264287 | KF264246 | KF264327 | KF774333 | KF774373 | KF774427 | KF264365 | KF264401 |
| *Amphiprion* | *latifasciatus* | GA083 | Madagascar | | KF264166 | KF264207 | KF264288 | KF264247 | KF264328 | KF774334 | KF774374 | KF774403 | KF264366 | |
| *Amphiprion* | *leucokranos* | GA066 | Solomon | 1 | KF264167 | KF264208 | KF264289 | KF819377 | | | | | | |
| *Amphiprion* | *leucokranos* | GA066 | Solomon | 2 | | | | KF819373 | | | | | | |
| *Amphiprion* | *mccullochi* | GA056 | Australia | | KF264168 | KF264209 | KF264290 | KF264249 | KF264330 | KF774335 | KF774376 | KF774414 | KF264368 | |
| *Amphiprion* | *melanopus* | GA012 | Bali | | KF264169 | KF264210 | KF264291 | KF264250 | KF264331 | KF774336 | KF774377 | KF774421 | KF264369 | KF264403 |
| *Amphiprion* | *nigripes* | GA055 | Sri Lanka | | KF264170 | KF264211 | KF264292 | KF264251 | KF264332 | KF774337 | KF774378 | KF774405 | KF264370 | KF264404 |
| *Amphiprion* | *ocellaris* | GA009 | Bali | | KF264171 | KF264212 | KF264293 | KF264252 | KF264333 | KF774338 | KF774379 | KF774428 | KF264371 | KF264405 |
| *Amphiprion* | *ocellaris* | GA021 | Bali (mocha) | | KF264172 | KF264213 | KF264294 | KF264253 | KF264334 | KF774339 | KF774380 | KF774429 | KF264372 | KF264406 |
| *Amphiprion* | *omanensis* | GA051 | Oman | | KF264173 | KF264214 | KF264295 | KF264254 | KF264335 | KF774340 | KF774381 | KF774404 | KF264373 | KF264407 |

| | | | | | | | | | | | | | |
|---|---|---|---|---|---|---|---|---|---|---|---|---|---|
| *Amphiprion* | *pacificus* | GA069 | Fiji | KF264174 | KF264215 | KF264296 | KF264255 | KF264336 | KF774341 | KF774382 | KF774399 | KF264374 | KF264408 |
| *Amphiprion* | *percula* | GA017 | wild picasso | KF264175 | KF264216 | KF264297 | KF264256 | KF264337 | KF774342 | KF774383 | KF774430 | KF264375 | KF264409 |
| *Amphiprion* | *percula* | GA039 | PNG | KF264176 | KF264217 | KF264298 | KF264257 | KF264338 | KF774343 | KF774384 | KF774432 | KF264376 | KF264410 |
| *Amphiprion* | *percula* | GA053 | Solomon Island | KF264177 | KF264218 | KF264299 | KF264258 | | KF774344 | KF774385 | KF774431 | KF264377 | |
| *Amphiprion* | *perideraion* | GA015 | Bali | KF264178 | KF264219 | KF264300 | KF264259 | KF264339 | KF774345 | KF774386 | KF774409 | KF264378 | KF264411 |
| *Amphiprion* | *perideraion* | GA020 | PNG | KF264179 | KF264220 | KF264301 | KF264260 | KF264340 | KF774346 | KF774387 | KF774408 | KF264379 | KF264412 |
| *Amphiprion* | *polymnus* | GA019 | Bali | KF264180 | | KF264302 | KF264261 | KF264341 | KF774347 | KF774388 | KF774422 | | KF264413 |
| *Amphiprion* | *polymnus* | GA038 | PNG | KF264181 | KF264221 | KF264303 | KF264262 | KF264342 | KF774348 | KF774389 | KF774423 | KF264380 | KF264414 |
| *Amphiprion* | *rubrocinctus* | GA070 | Australia | KF264182 | KF264222 | KF264304 | KF264263 | KF264343 | KF774349 | KF774390 | KF774419 | KF264381 | KF264415 |
| *Amphiprion* | *sandaracinos* | GA018 | Bali | KF264183 | KF264223 | KF264305 | KF264264 | KF264344 | KF774350 | KF774391 | KF774397 | KF264382 | KF264416 |
| *Amphiprion* | *sandaracinos* | GA037 | PNG | KF264184 | KF264224 | KF264306 | KF264265 | KF264345 | KF774351 | KF774392 | | | KF264417 |
| *Amphiprion* | *sebae* | GA029 | Sri Lanka | KF264185 | KF264225 | KF264307 | KF264266 | KF264346 | KF774352 | | | KF264383 | KF264418 |
| *Amphiprion* | *tricinctus* | GA058 | Marshall Islands | KF264186 | KF264226 | KF264308 | KF264267 | KF264347 | KF774353 | KF774393 | KF774413 | KF264384 | KF264419 |
| *Premnas* | *biaculeatus* | GA008 | Bali (white stripe) | KF264187 | KF264227 | KF264309 | KF264268 | KF264348 | KF774354 | KF774394 | KF774433 | KF264385 | KF264420 |
| *Premnas* | *biaculeatus* | GA025 | Malaysia (yellow stripe) | KF264188 | KF264228 | KF264310 | KF264269 | KF264349 | KF774355 | KF774395 | KF774435 | KF264386 | KF264421 |
| *Premnas* | *biaculeatus* | GA065 | East Malaysia (green spot) | KF264189 | KF264229 | KF264311 | KF264270 | | KF774356 | | KF774434 | KF264387 | |
| *Abudefduf* | *saxatilis* | | | AY279673 | AY208410 | AY208553 | FJ616840 | JX188794 | KC825917 | | KC830145 | FJ616621 | JX189122 |
| *Chromis* | *cyanea* | | | AF285947 | AY208384 | AY208529 | | JX188795 | | | | AY208639 | JX189123 |
| *Chrysiptera* | *rex* | | | FJ616440 | AY208438 | AY208572 | FJ616879 | | | | | JN935826 | |
| *Neopomacentrus* | *azysron* | | | JF457542 | | JF458181 | FJ616904 | | | | | FJ616684 | |
| *Pomacentrus* | *amboinensis* | | | FJ616474 | | | FJ616913 | | | | | JN935821 | |
